# High Transmembrane Voltage Raised by Close Contact Initiates Fusion Pore

**DOI:** 10.3389/fnmol.2016.00136

**Published:** 2016-12-09

**Authors:** Bing Bu, Zhiqi Tian, Dechang Li, Baohua Ji

**Affiliations:** ^1^Biomechanics and Biomaterials Laboratory, Department of Applied Mechanics, Beijing Institute of TechnologyBeijing, China; ^2^Key Laboratory of Biomedical Information Engineering of Ministry of Education, Center for Mitochondrial Biology and Medicine, School of Life Science and Technology, Xi'an Jiaotong UniversityXi'an, China

**Keywords:** membrane fusion, transmembrane voltage, fusion pore formation, exocytosis, mechanical force

## Abstract

Membrane fusion lies at the heart of neuronal communication but the detailed mechanism of a critical step, fusion pore initiation, remains poorly understood. Here, through atomistic molecular dynamics simulations, a transient pore formation induced by a close contact of two apposed bilayers is firstly reported. Such a close contact gives rise to a high local transmembrane voltage that induces the transient pore formation. Through simulations on two apposed bilayers fixed at a series of given distances, the process in which two bilayers approaching to each other under the pulling force from fusion proteins for membrane fusion was mimicked. Of note, this close contact induced fusion pore formation is contrasted with previous reported electroporation under *ad hoc* applied external electric field or ionic charge in-balance. We show that the transmembrane voltage increases with the decrease of the distance between the bilayers. Below a critical distance, depending on the lipid composition, the local transmembrane voltage can be sufficiently high to induce the transient pores. The size of these pores is approximately 1~2 nm in diameter, which is large enough to allow passing of neurotransmitters. A resealing of the membrane pores resulting from the neutralization of the transmembrane voltage by ions through the pores was then observed. We also found that the membrane tension can either prolong the lifetime of transient pores or cause them to dilate for full collapse. This result provides a possible mechanism for fusion pore formation and regulation of pathway of fusion process.

## Introduction

Membrane fusion, as an important and ubiquitous cellular process, lies at the heart of neuronal communication wherein neurotransmitters are quickly released from synaptic vesicles following fusion with the presynaptic membrane. During this process, an initial fusion pore is assumed to be created between the two apposed membranes, and this tiny pore may either expand to a larger one or close back, which leads to two kinds of fusion mode (Alabi and Tsien, 2013). One type of fusion mode called full fusion (FF) requires fusion pore expand to the point where the vesicle membrane flattens into the plasma membrane surface, leading to complete luminal content release(Chernomordik and Kozlov, 2003; Jackson and Chapman, 2008); the other type of fusion mode proceeds without pore expansion, where vesicles transiently fuse at the plasma membrane to release a part of their neurotransmitters (Richards, 2009) without full collapse into the plasma membrane known as “kiss-and-run” fusion (KR) (He and Wu, 2007; Zhang et al., 2009). Although, the experimental results on membrane fusion at synapses are mounting (He and Wu, 2007), it is difficult to directly image the fusion process at sufficient resolution, thus the molecular mechanism of the fusion initiation is still unclear.

In addition, it is also unclear how the fusion process is regulated *in vivo*. For instance, there is a long standing debate over FF and KR fusion for the regulating mechanisms behind the synaptic release (Marx, 2014). One of the important issues in the debate is an incomplete understanding of the structural mechanics of the membrane under the force of fusogenic proteins (Jahn et al., 2003; Rizo and Rosenmund, 2008), and how these forces act on the membrane during the fusion process. It is known that when a small membrane pore is created (Chernomordik and Kozlov, 2008), it might either close or expand (Chanturiya et al., 1997; Wong et al., 2007; Diao et al., 2012). The reversible fusion pore modality, corresponding to KR fusion, enables a rapid and economical vesicle recycling compared to FF that may require ancillary proteins to retrieve fused vesicles (He and Wu, 2007). However, how the fusion pore is regulated for the fast synaptic transmission and the exact molecular picture of the pore evolution remains obscure.

Transient pore formation has been observed in many *in vivo* and *in vitro* systems, such as liposomes from yeast vacuoles (Starai et al., 2007; Zucchi and Zick, 2011), mating yeast pairs (Aguilar et al., 2007), cell pairs mediated by influenza fusase hemagglutinin (HA) (Blumenthal and Morris, 1999; Frolov et al., 2003), and proteoliposomes reconstituted with neuronal SNAREs (SNAP [Soluble NSF attachment protein] Receptors) (Dennison et al., 2006; Lai et al., 2013; Gong et al., 2015). And there are also many proposed mechanisms for the opening of the membrane pores through *in silico* simulations, such as transmembrane ionic charge imbalance, external electric and mechanical forces (Müller et al., 2003; Gurtovenko et al., 2010). However, the questions of how transient pores are produced in physiological conditions, and how they are regulated remain enigmatic. Here, through full atom molecular dynamics (MD) simulations, we discovered a fast and transient membrane pore formation when the distance of two apposed bilayers was set below a threshold value. The average size of these pores was of 1~2 nm in diameter, which is large enough for passing neurotransmitters. The initial pore could either shrink or expand depending on the magnitude of membrane tension. The high local transmembrane voltage caused by the close contact of apposed membranes was responsible for the formation of these fast and transient membrane pores. This study might thus shed promising light on the molecular mechanisms of pore formation and evolution.

## Methods

### MD simulation

**Figure 1:**
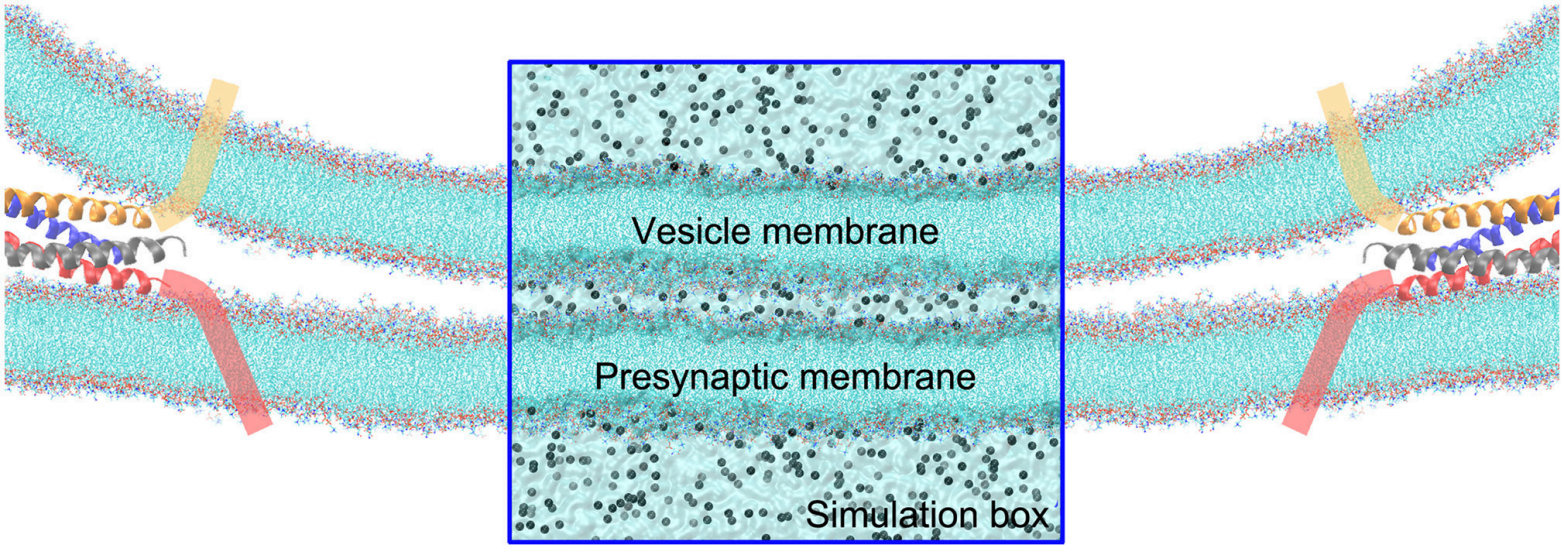
**Illustration of the contact of a vesicle with the presynaptic membrane in a close proximity**. The tails of lipids are shown by cyan lines, and ions by gray balls. The fusion protein shown as cartoon α-helices, are assumed to pull the vesicle to approach to the synaptic membrane. The rectangular zone is the simulation box, where the water molecules are shown by transparent surface. The simulation box contains five layered regions—form top to bottom is: the vesicle cytosol, vesicle membrane, cellular cytosol between the vesicle and presynaptic membranes, presynaptic membrane, and the interstitial fluid around the synapse. For the sake of clarity, water molecules are not explicitly shown outside the simulation box.

To simulate the pore formation at the contact of a vesicle with the synaptic membrane, we created two apposed lipid bilayers set at various distances (see Figure 1). The lipid bilayers were generated by Membrane Builder online service (Jo et al., 2008, 2009). The simulation system was set up following a similar procedure in our previous studies (Li et al., 2010, 2011, 2015; Lai et al., 2015; Xu et al., 2015). The system was solvated in a ~23 × 23 × 20 nm^3^ TIP3P (Jorgensen et al., 1983) water box, with ~210,000 water molecules and potassium ions added to neutralize the system. All the simulations were performed using GROMACS package (Van Der Spoel et al., 2005) with CHARMM36 force field (Klauda et al., 2010). Periodic boundary condition was applied and the temperature was coupled to 300 K with V-rescale algorithm (Bussi et al., 2007). The pressure was coupled to 1 bar with Parrinello-Rahman approach (Parrinello and Rahman, 1981). The LINCS algorithm (Hess, 2008) was applied to constrain the covalent bonds with H-atoms. The time step of the simulations is 2.0 fs. The cut-off of the non-bonded interactions was set to 10 Å. The particle mesh Ewald (PME) (Essmann et al., 1995) method was used to calculate the long-range electrostatic interactions. The non-bonded pairs were updated in every 10 steps. Each MD simulation was performed independently for three replicates. The simulation system was validated by its reproducing the stalk-like structure in experiments under dehydration condition (Yang and Huang, 2002). All graphics and visualization analysis were processed using the VMD program (Humphrey et al., 1996).

Here the lipid composition was adopted as Chol:DOPC:POPE:POPS with different ratio of percentage as (20:45:20:15%), (20:48:20:12%), (20:51:20:9%), (20:54:20:6%), and (20:55.5:20:4.5%). Chol, DOPC, POPE, POPS are abbreviation of cholesterol, 1,2-dioleoyl-sn-glycero-3-phosphocholine, 1-palmitoyl-2-oleoyl-sn-glycero-3-phosphoethanolamine, and 1-palmitoyl-2-oleoyl-sn-glycero-3-phenylserine, respectively.

In the molecular dynamics simulations, we carried out simulations on two bilayers set at various membrane distances, where the distance D was chosen by reducing its value from 6 nm, in 0.5 nm steps (see Figure S1), for mimicking the approaching process of two apposed bilayer under the pulling force of fusion protein for membrane fusion. Each simulation was done at a given constant membrane distance.

The ions were uniformly distributed in the three cytosol/fluid regions, i.e., the vesicle cytosol, cellular cytosol between the vesicle and presynaptic membranes, and the interstitial fluid around the synapse, as shown in Figure S1. The ionic concentration (potassium) was about 0.04~0.15 mol/L, depending on the lipid composition and their percentage. The density of water was 1 g/cm^3^. Note that for specific lipid composition, the density of water and ions was kept constant. In the simulation, we chose potassium ions as the only ions in the system without chloride ions. For double check, we also did simulations with both potassium and chloride ions. In the presence of chloride ions, we put more potassium ions to neutralize the system. Our results showed that they had similar results as those in the absence of chloride ions.

For simulating the pore evolution under membrane tension, we built membrane tension Γ by applying pressure on the simulation box, given by Γ = *L_Z_*(*P_Z_* − *P_Lat_*), where *L_Z_* is the length of the simulation box in the Z direction, *P_Z_* is the pressure along the Z direction, and *P_Lat_* is the pressure along lateral direction in the membrane plane (Leontiadou et al., 2004).

### Calculation of the transmembrane voltage

To calculate the transmembrane voltage in MD simulations, we adopted the procedure proposed by Tieleman by solving a one dimensional Poisson's equation (Tieleman, 2004). Firstly, the charge density along the Z direction ρ(*Z*) was calculated by averaging the net charges over the membrane plane. The electric potential can be obtained by solving the one dimensional Poisson's equation, i.e., $\Phi \left(Z\right)=-\frac{1}{\varepsilon }\overset{Z}{\underset{0}{\int }}\overset{Z\prime }{\underset{0}{\int }}\rho \left(Z\prime \prime \right)dZ\prime \prime dZ\prime +\Phi \left(0\right)$, where Φ(0) was chosen as zero at the axis of symmetry of the system at Z = 0. The difference of the electric potential through the membrane gives the transmembrane voltage.

### Theoretical model of electric potential

**Figure 2:**
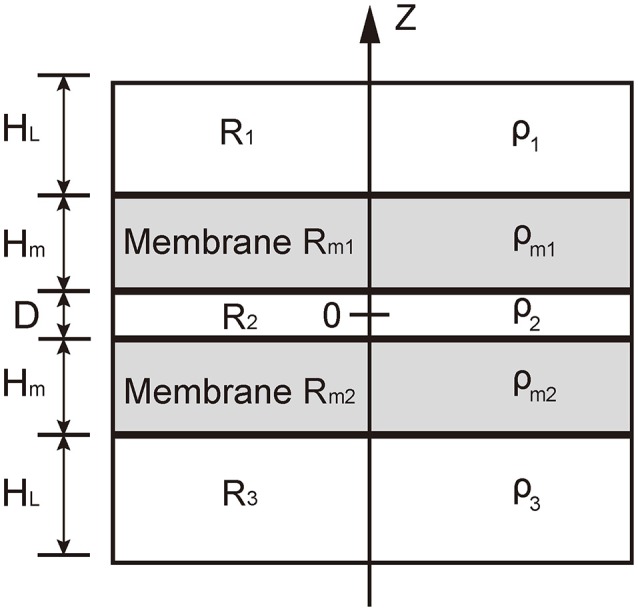
**Illustration of the theoretical model**. The domain *R*_1_ represents the cytosol in vesicle, *R*_2_ represents the cytosol between the vesicle and the presynaptic membranes, and *R*_*m*1_ represents interstitial fluid around synapse. *R*_*m*1_ and *R*_*m*2_ represents the vesicle membranes and presynaptic membrane, respectively. The charge density in domains *R*_1_ and *R*_3_ are denoted by ρ_1_ and ρ_3_, respectively; that in domain *R*_2_ by ρ_2_, and that in the domains *R*_*m*1_ and *R*_*m*2_ by ρ_*m*1_ and ρ_*m*2_, respectively.

In our model, we used five layers to consider the five regions at the contact between the vesicle and presynaptic membrane: the cytosol in vesicle, *R*_1_, the vesicle membrane *R*_*m*1_, the cellular cytosol between vesicle and presynaptic membrane, *R*_2_, the presynaptic membrane *R*_*m*2_, and the interstitial fluid around synapse, *R*_3_ (see Figure 2). Since we considered the local contact region between vesicle and presynapse, we assumed that the distribution of charges was uniform in each layer. For a neutralized system, the charge density should satisfy

(1) $$\begin{array}{l} \rho_{1}H_{L}+\rho_{3}H_{L}+\rho_{2}D+\rho_{m1}H_{m}+\rho_{m2}H_{m}=0 \end{array}$$

where ρ_1_, ρ_2_, ρ_3_, ρ_*m*1_, and ρ_*m*2_ are charge density in layers *R*_1_, *R*_2_, *R*_3_, *R*_*m*1_, and *R*_*m*2_, respectively. *H*_*L*_ is the thickness of layers *R*_1_ and *R*_3_ (here we adopted that the thickness of the two domains is identical), *H*_*m*_ is the thickness of membrane, and D is the distance between the two apposed membranes. In this situation, the electric potential satisfies the one dimensional Poisson-Boltzmann equation,

(2) $$\begin{array}{l} \frac{d^{2}\Phi \left(Z\right)}{dZ^{2}}=-\frac{\rho \left(Z\right)}{\varepsilon } \end{array}$$

where Φ(*Z*) is the electric potential along the *Z* direction, ρ(*Z*) is the charge density and ε the dielectric constant. We set ε = 3 according to experimental measurement by Gramse et al. (2013). And we obtained the electric potential as

(3) $$\begin{array}{l} \Phi \left(Z\right)=-\frac{1}{\varepsilon }\overset{Z}{\underset{0}{\int }}\overset{Z\prime }{\underset{0}{\int }}\rho \left(Z\prime \prime \right)dZ\prime \prime dZ\prime \end{array}$$

The result of the integral in Equation (3) can be found in Supplementary Material. Thus the transmembrane voltage of the vesicle membrane can be calculated as

(4) $$\begin{array}{l} \Delta V_{1}=-\frac{1}{\varepsilon }\text{(}\frac{1}{2}\rho_{m1}H_{m}+\rho_{m2}H_{m}+\rho_{2}D+\rho_{3}H_{L}\text{)}H_{m} \end{array}$$

and the corresponding voltage of the presynaptic membrane is

(5) $$\begin{array}{r} \Delta V_{2}=-\frac{1}{\varepsilon }\left(\frac{1}{2}\rho_{m2}H_{m}+\rho_{m1}H_{m}+\rho_{2}D+\rho_{1}H_{L}\right)H_{m} \end{array}$$

As shown in Equations (4 and 5), because ρ_*m*1_ and ρ_*m*2_ are negative, Δ*V*_1_ and Δ*V*_2_ linearly increase with the reduction of the inter-membrane distance *D*. If we assume ρ_1_ = ρ_3_, and ρ_*m*1_ = ρ_*m*2_ = ρ_*m*_, we have

(6) $$\begin{array}{r} \Delta V=\Delta V_{1}=\Delta V_{2}=-\frac{1}{2\varepsilon }\left(\rho_{m}H_{m}+\rho_{2}D\right)H_{m} \end{array}$$

## Results

### Close contact induces transient pore formation

**Figure 3:**
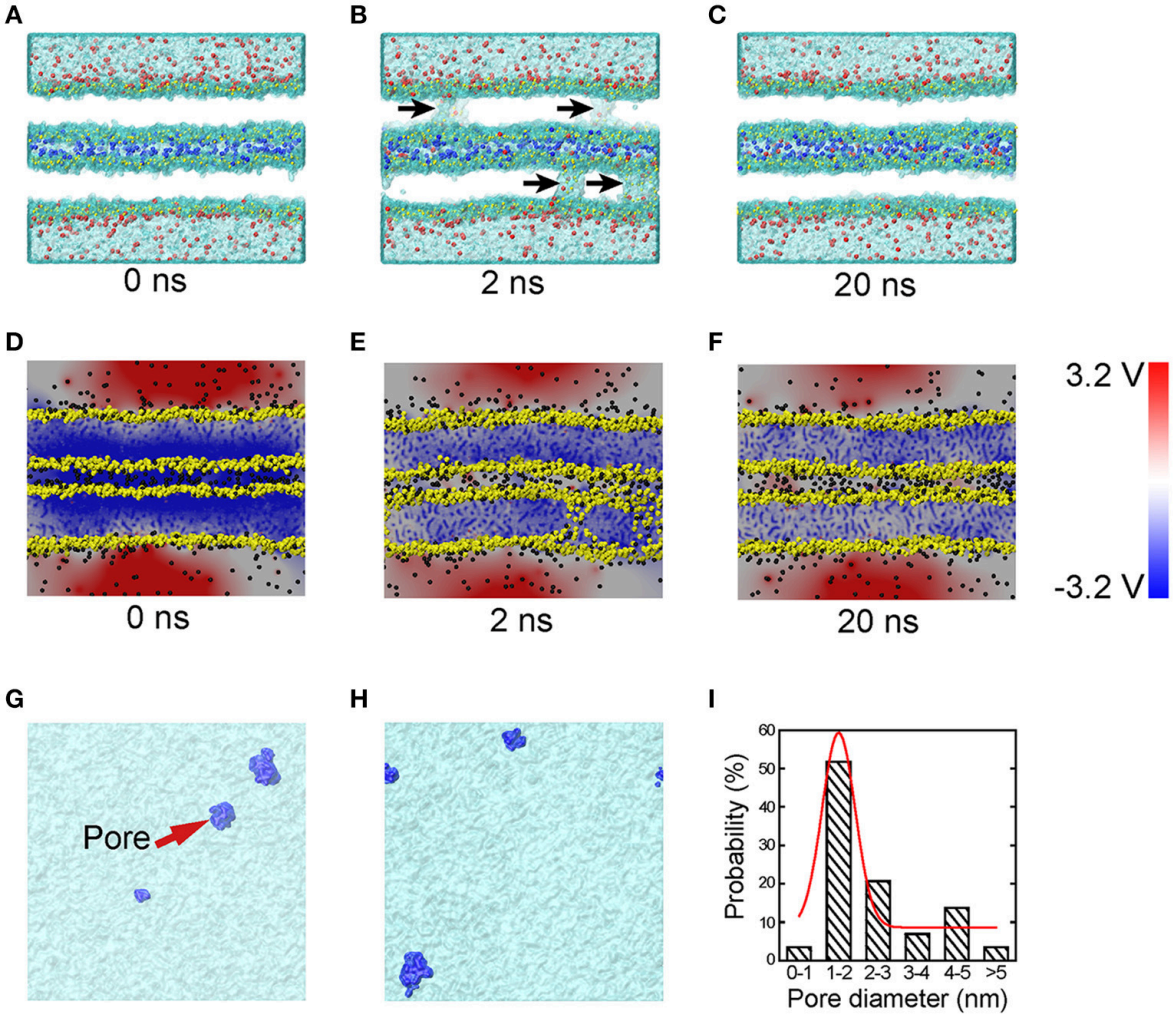
**MD simulations and corresponding electrical potential distributions of the sequential steps of close contact, pore formation and ion leakage, and membrane healing**. Snapshots of MD simulations **(A–C)** depict the formation of transient fusion pores. The vesicle and presynaptic membranes are brought into close apposition at 0 ns **(A)**, at 2 ns **(B)** pores form and there is ion leakage, and at 20 ns **(C)** membrane healing. The arrows in **(B)** indicate transient pores. To clearly show the ion transportation, the ions between the two membranes are shown in blue, while those in the vesicle cytosol and the interstitial fluid are shown in red. **(D–F)** Snapshots of the distribution of electric potential at the condition of close contact **(D)**, pore formation and ion exchange **(E)**, and membrane healing **(F)**. Top-down snapshots of the transient pores in the two membranes **(G,H)** with pores shown in blue. **(I)** The size distribution of the transient pores, where the red line is Gaussian curve. The heads of lipids are shown in yellow, and the tails of the lipids are not shown for a clear representation of the pores. The water molecules are shown as transparent surface.

To understand the very early stage of membrane fusion when the vesicle and presynaptic membranes get close, we simulated two apposed membranes fixed at a series of given distances using MD simulations, mimicking the docking process pulled by SNAREs or other accessory proteins, as shown in Figure 1. By using a smaller distance between membranes below a critical value (discussed below), we found the formation of tiny pores on the membranes (Figure 3). When the two membranes were within ~5 nm, a water line first formed across the thickness of membrane in several nanoseconds, followed by the formation of a transient pore, like a water channel, spanning the membranes (Figure 3B). Then the transient pore grew rapidly, causing considerable redistribution of lipid headgroups close to the channel. At the same time, ions can transport through these pore, as shown in Figure 3B. The duration of the ion transportation was very short (approximately 10 ns). Once the ion transportation was finished, the pores healed automatically as the results of balancing charge difference (Figure 3C). More details of the pore formation, ions leakage and pore healing can be found in Figure S2 and Movie S1 in Supplementary Material. To examine the physiological relevance of our simulations, the membrane distance was further reduced to mimic a higher degree of dehydration, which induced a stalk-like structure for hemifusion. This result is consistent with previous experiments of Yang and Huang (Yang and Huang, 2002) (see Figure S3) and validates our MD simulations.

To study the molecular mechanisms of the pore formation, we calculated the electric potential in our system. Our results showed that the distribution of electric potential is highly dependent on inter-membrane distance. As the distance between the two membranes decreased, the difference of electric potential across the lipid bilayer, i.e., the transmembrane voltage, rose significantly (Figure 3D). This transmembrane voltage formed the driving force for the pore formation, consistent with earlier reports that the transmembrane voltage caused by ionic charge imbalance between the two sides of the membrane (Gurtovenko and Vattulainen, 2005), or by external electric field (Tieleman, 2004; Sun et al., 2011), induces fusion pore and ion trafficking. Meanwhile, in previous studies, the transmembrane voltage was ad-hoc applied on the membrane (Tieleman, 2004; Gurtovenko and Vattulainen, 2005; Sun et al., 2011). Here we found that without the above *ad hoc* conditions, reducing the membrane distance alone was sufficient to cause a dramatic increase in transmembrane voltage capable of producing the driving force necessary for the fusion pore formation. Our simulation showed that the transmembrane voltage for pore formation produced by the close contact is in the range of 0.7~1.2 V, consistent with the value of 0.5~1.5 V used in electroporation experiments (Weaver and Chizmadzhev, 1996). The mean size of the pores was round 1–2 nm (see Figures 3G–I), which is large enough for the passing of small neurotransmitters (Zhang et al., 2009).

### Transmembrane voltage vs. the membrane distance

**Figure 4:**
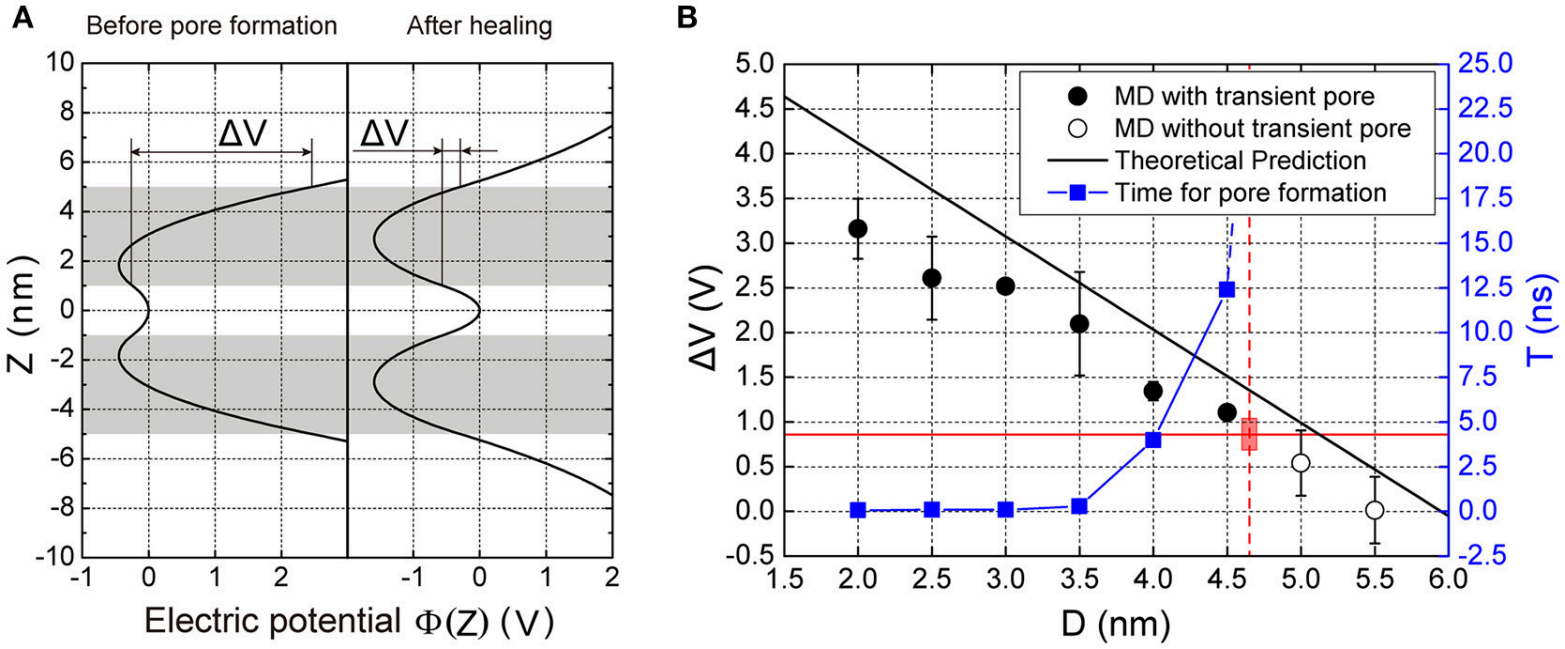
**Electric potential and transmembrane voltage of the membranes. (A)** The predicted electric potential before pore formation and after pore closing. Δ*V* is the transmembrane voltage of the vesicle. **(B)** The transmembrane voltage and the time for pore formation, T as function of the membrane distance, D. The black solid dots are results of MD simulations denoting the voltage that enables pore formation, while the hollow dots are for those where pore formation did not occur. The black line is the theoretical prediction of transmembrane voltage according to our model (see Equation 6 in Methods). The red horizontal solid line indicates the critical transmembrane voltage (Δ*V*_*critical*_) required for pore formation. The blue solid square indicates the time needed for pore formation calculated from MD simulations. The vertical red dashed line indicates the critical distance for pore formation (4.7 nm), beyond which the time for the pore formation is infinite. Here the lipid composition was Chol:DOPC:POPE:POPS (20:45:20:15%). Chol, DOPC, POPE, POPS are abbreviation of cholesterol, 1,2-dioleoyl-sn-glycero-3-phosphocholine, 1-palmitoyl-2-oleoyl-sn-glycero-3-phosphoethanolamine, and 1-palmitoyl-2-oleoyl-sn-glycero-3-phenylserine, respectively. Each MD simulation was performed independently for three replicates, each for 20 ns.

To understand the mechanism of transmembrane voltage rising with the decrease of membrane distance, we employed a physical model to derive the field of electric potential in the apposed membranes as a function of the membrane distance (Figure 2). For a given ion distribution corresponding our MD simulations, we obtained the analytical solution of the field of electric potential and in particular, a linear relationship between the transmembrane voltage and the membrane distance for a close contact (see Methods). We found that when the membrane distance was less than ~5 nm, the profile of the electric potential in each membrane became severely asymmetric with respect to the central line of the lipid bilayer thus producing a high transmembrane voltage (Figure 4A). However, after the ion leakage, the profile of the electric potential almost resumed its symmetry, resulting in a reduced transmembrane voltage (Figures 3F, 4A). This prediction is consistent with observations from our MD simulations (Figure S4). Moreover, it clearly indicates that the transmembrane voltage, produced by the close contact of the apposed membranes, was the driving force for the transient pore formation.

### Threshold values of membrane distance

To find the threshold value of membrane distance for the pore formation, we performed a series of MD simulations at different membrane distances. We defined the critical distance as the distance above which the pore formation will not happen in up to 20 ns in our simulation (Figure 4B). When the distance, D, was larger than a critical value (~5 nm), the voltage was nearly zero because of the symmetry of the field of electric potential in the membrane. But as the distance decreased, the voltage increased, being negatively proportional to the distance (see Equation 6 in Methods). Referring to our MD simulations, a series of membrane distance D was chosen by reducing its value from 6 nm, in 0.5 nm steps, for the lipid composition Chol:DOPC:POPE:POPS (20:45:20:15%). In these settings, we found that the transmembrane voltage produced by the close contact could be up to >3 V, consistent with previous results produced by the transmembrane ionic charge imbalance (Gurtovenko and Vattulainen, 2005). We found that when the distance was larger than 5 nm, there was no transient pore formation. But when D became smaller, pore formation could be observed. In addition, the smaller the distance is, the faster the pore could form (Figure 4B, blue line). These results suggest there is a threshold value for the inter-membrane distance required for the transient pore formation.

### Effect of lipid composition on fusion pore formation threshold

**Figure 5:**
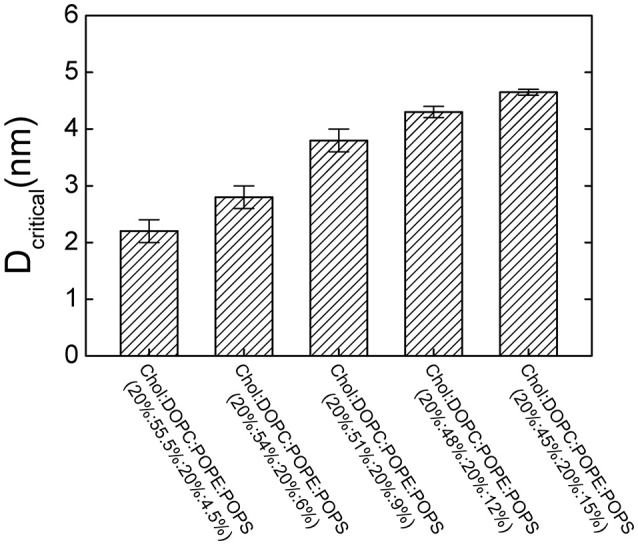
**The critical membrane distance (*D*_*critical*_) for pore formation at different lipid compositions**. *D*_*critical*_ increases with increasing the percentage of POPS.

To investigate the contribution of the lipid composition in fusion pore formation, we modified our simulations to adopt various lipid compositions by changing the percentage of POPS, which modulates the charge density on the membrane. Our results showed that the threshold value of membrane distance for pore formation was highly dependent on the percentage of POPS (Figure 5). For example, we found that reducing the percentage of POPS in both membranes generally reduced the threshold value due to dissipated charge density of the membrane. The lower the percentage of the POPS, the smaller the critical distance needed, and thus the more difficult the pore formation. This result also emphasizes why two membranes should be brought close together for fusion: only a membrane distance smaller than the threshold value can produce a sufficiently high transmembrane voltage that can induce pore formation.

Note that we did not consider the asymmetry of lipid composition between the inner and outer leaflet of cell membrane (Svennerholm, 1968; Devaux, 1991). Although, the asymmetry may affect the process of fusion pore formation, it is a secondary factor in the membrane fusion in comparison with the close contact between the membranes. Heterogeneity of lipid composition may also be an influencing factor in the fusion process. For instance, phosphatidylinositol-4,5-bisphosphate (PIP2), a lipid with -5e negative charges, is an important composition that accumulates at the location of fusion site (McLaughlin and Murray, 2005; Graber et al., 2014). But the localized PIP2 mainly interacts with syntaxin proteins during the course of membrane fusion. And the function of fusion protein syntaxin is to pull the two apposed membranes to be close contact, which we had effectively considered by applying pulling force on the membranes. To test the influence of the asymmetric lipid composition and localized PIP2, we performed an additional simulation with 5% PIP2 in the cytoplasmic leaflet which make the two membranes asymmetric, as shown in Figure S5. It shows that the close contact of the two membranes containing localized PIP2 could still give rise to a high local transmembrane voltage which induced the pore formation.

### Force regulates the pore evolution

**Figure 6:**
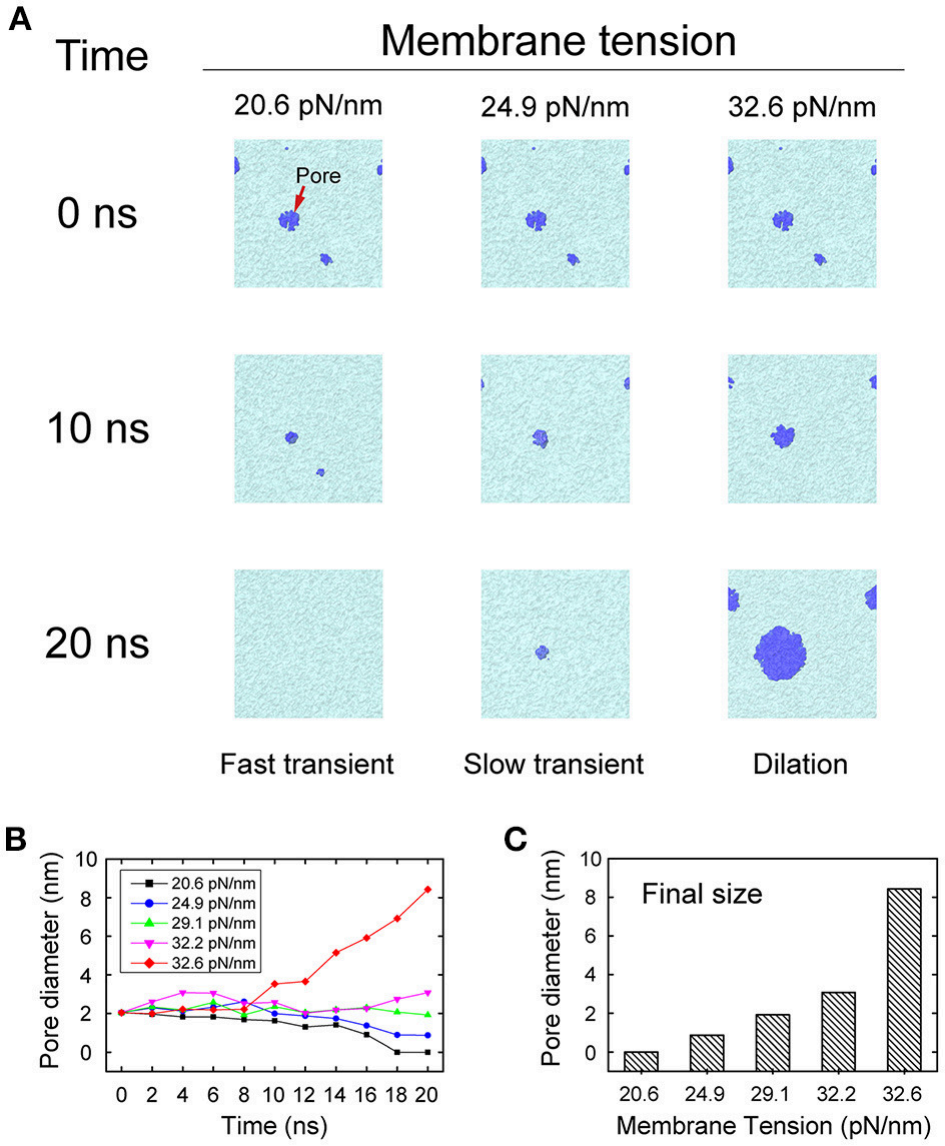
**Evolution of fusion pores under membrane tension. (A)** Snapshots of the evolution of fusion pores under various degrees of membrane tension. A small membrane tension (left column) of 20.6 pN/nm allows quick resealing of the pores in 20 ns, which leads to fast transient pores. A medium membrane tension (middle column) of 24.9 pN/nm stabilizes the pores and prolongs the duration of the transient pores. Finally, a large membrane tension (right column) of 32.6 pN/nm leads to dilation of the pores. The lipids of membrane are represented by cyan, while the fusion pores are represented by blue. **(B)** The changing of the pore size as function of the simulation time at different membrane tension. **(C)** The final size of fusion pore at *t* = 20 ns.

During membrane fusion, fusion proteins apply not only vertical force to pull the vesicle close to the presynaptic membrane, but also lateral force along the membrane which produces a local membrane tension. To mimic this behavior of fusion proteins, we imposed a lateral force on the membrane once the pores were initiated, to study how the local membrane tension regulates its structural evolution. We found that when the tension was small (20.6 pN/nm), the membrane pore shrank and resealed quickly in less than 20 ns. However, when the tension was increased (24.9 pN/nm), the resealing process of the fusion pore became slower. When the tension was further increased (32.6 pN/nm) the fusion pore started to expand and the vesicle began to collapse into the membrane (Figure 6). In our simulations, the first two cases, where the membranes resealed, are correspond to the scenarios of fast and slow transient pore for KR fusion, while the third case corresponds to the pore dilation of FF. This result clearly indicates that the stability and evolution of the fusion pore, and the selection of fusion pathways between KR fusion and FF, could be regulated by the membrane tension.

## Discussion

This study suggests a feasible mechanism for pore formation in membrane fusion: a close contact of membranes could generate a high transmembrane voltage, which in turn produces the membrane pores. Note that the close-contact induced voltage observed in our simulation is contrasted with the *ad hoc* applied voltage in previous electroporation experiments (Weaver and Chizmadzhev, 1996). The opening of the fusion pore, initiated from a voltage-dependent perturbation of lipid organization at the contact of apposed bilayers, begins with a lateral parting of headgroups on two membranes for allowing water molecules to enter the hydrophobic regions (Helm et al., 1992; Fesce et al., 1994). Although, the transmembrane voltage can be produced by an *ad hoc* ionic charge imbalance across membranes (Gurtovenko and Vattulainen, 2005) or external electric field (Tieleman, 2004; Sun et al., 2011), how cells modulate the ionic charge imbalance or electric fields in order to produce the required voltage is not known. In this study, we showed that the transmembrane voltage can be generated by reducing the distance between two membranes, an action that could be carried out by fusion proteins, such as SNAREs or the calcium sensor, synaptotagmin, etc., (Diao et al., 2009, 2013; Kyoung et al., 2013; Lai et al., 2014). Our data agrees with a growing body of work suggesting that pore formation, stability, and evolution can be subject to regulation by cellular processes rather than a stochastic thermodynamic process (Wang et al., 2009; Risselada and Grubmüller, 2012).

**Figure 7:**
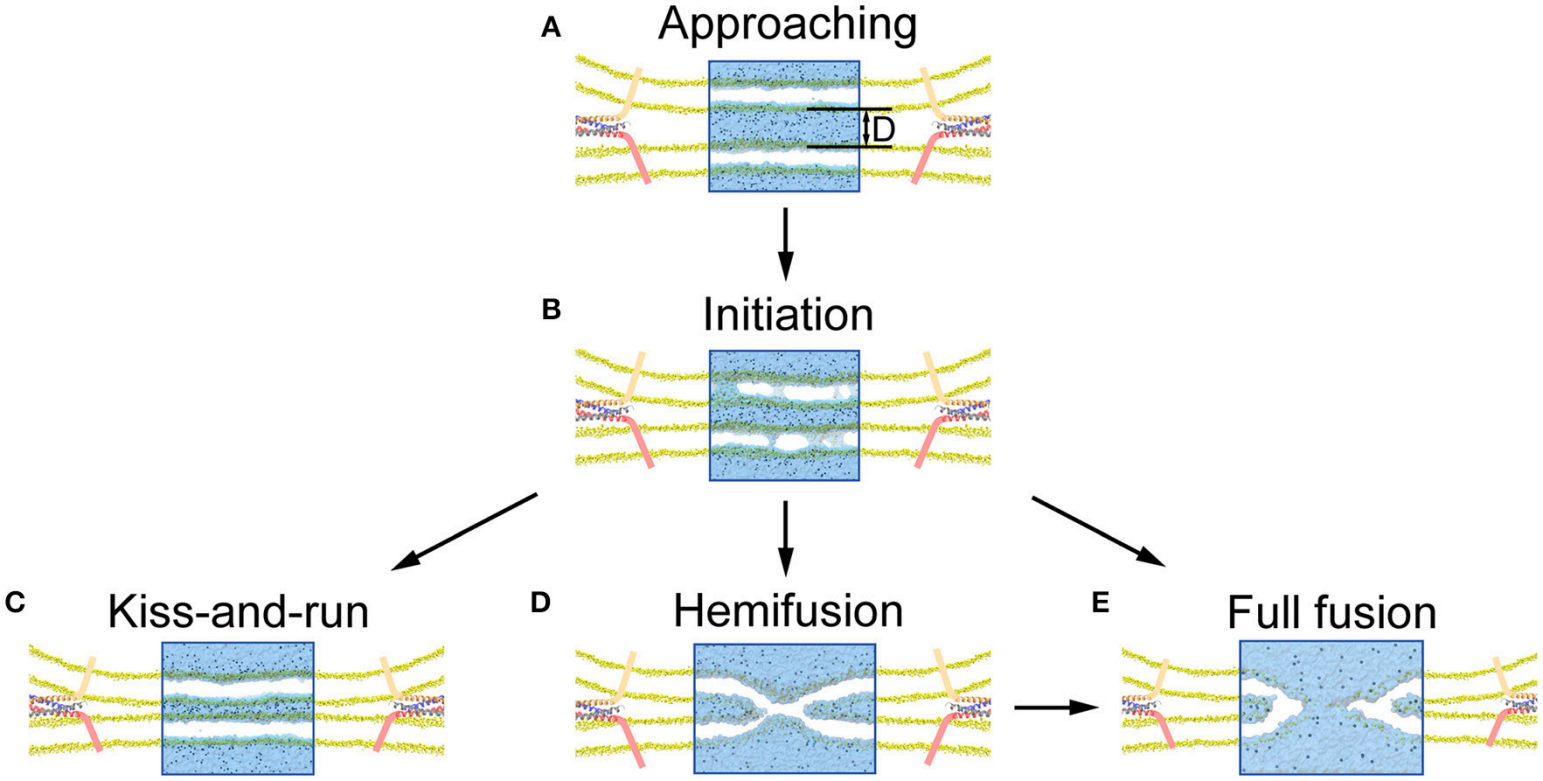
**Illustration of pore formation, evolution and selection of fusion mode proposed by our MD simulation. (A)** Close contact of vesicle with presynaptic membrane pulled by the fusion proteins. **(B)** Pore formation and associated release of neurotransmitters once the membrane distance, *D*, is smaller than a critical value. **(C)** Resealing of the fusion pores after the release of neurotransmitters for a KR fusion. **(D)** Formation of stalk-like structure for hemifusion appears when the membrane distance is further reduced. **(E)** Full fusion can be achieved by either expansion of the fusion pore (**B** under a larger lateral force), or following the hemifusion **(D)**.

In agreement with previous studies, our results further support the notion that native proteins are capable of modulating fusion pore dynamics with additional helps from other proteins producing differential layers of modulation (Alabi and Tsien, 2013; Lai et al., 2015). We found that the fusion pore dynamics appeared to be finely modulated by the type and magnitude of mechanical force exerted on membrane. A vertical force pulls the vesicle close to the plasma membrane, increasing the local voltage difference resulting in more pore formation. A further increase of the vertical force produces a closer contact for to a stalk-like structure for hemifusion (Figure S3), as observed in previous experiments (Yang and Huang, 2002; Zhao et al., 2016). In contrast, we found that the membrane tension at the contact site induced by the lateral force stabilizes pore formation and increases pore duration, while a larger force could induce pore expansion (Figure 6). These results suggest that the fusion dynamics could be regulated by the force of fusion proteins, and by extension, so as the rate of neurotransmitter diffusion out of the synaptic vesicle (Richards, 2009; Zhang et al., 2009; Alabi and Tsien, 2013). Since KR fusion and hemifusion are intermediate states between prime (un-fused) state and FF, the mechanical forces produced by fusion machinery proteins could select among KR, hemifusion, and FF (Figure 7), to modify the kinetics of neurotransmitter release which could ultimately provide a mechanism to regulate synaptic strength and achieve synaptic plasticity (He and Wu, 2007).

In conclusion, we provided a new mechanism for the initiation of fusion pore and its evolution through MD simulations of two membranes being set close together. Subsequent fusion pore initiation occurred within several nanoseconds once the membrane distance was smaller than ~5 nm. A high transmembrane voltage induced by close membrane proximity could cause these fast-forming transient membrane pores. The fusion pores are able to conduct ions across membrane and in doing so, alleviate the voltage difference which leads to final resealing of the membranes within a few nanoseconds. These sequential processes of pore formation, ion exchange, and pore healing in our MD simulations are consistent with the features of KR fusion observed in experiments (Zhang et al., 2009). In addition, we showed that the applied force on the membrane from native proteins plays a crucial role in modulating the stability and lifetime of fusion pores and may regulate the ultimate fusion mode to either KR fusion or FF. Therefore, both the pore formation and evolution for membrane fusion are tightly controlled by fusion proteins in physiological conditions.

## Author contributions

DL and BJ designed research; BB, ZT, and DL performed research; BB, ZT, DL, and BJ analyzed data; BB, ZT, DL, and BJ wrote the paper. All authors reviewed the manuscript.

## Funding

This paper was supported by 973 programs (2015CB856304) and Natural Science Foundation of China (Grant No. 11372042, 11221202, 11532009, and 11202026).

### Conflict of interest statement

The authors declare that the research was conducted in the absence of any commercial or financial relationships that could be construed as a potential conflict of interest.

## References

[B1] Aguilar P. S. Engel A. Walter P. (2007). The plasma membrane proteins Prm1 and Fig1 ascertain fidelity of membrane fusion during yeast mating. Mol. Biol. Cell 18, 547–556. 10.1091/mbc.E06-09-0776 17151357 PMC1783792

[B2] Alabi A. A. Tsien R. W. (2013). Perspectives on kiss-and-run: role in exocytosis, endocytosis, and neurotransmission. Annu. Rev. Physiol. 75, 393–422. 10.1146/annurev-physiol-020911-153305 23245563

[B3] Blumenthal R. Morris S. J. (1999). The influenza haemagglutinin-induced fusion cascade: effects of target membrane permeability changes. Mol. Membr. Biol. 16, 43–47. 10.1080/096876899294742 10332736

[B4] Bussi G. Donadio D. Parrinello M. (2007). Canonical sampling through velocity rescaling. J. Chem. Phys. 126, 014101. 10.1063/1.2408420 17212484

[B5] Chanturiya A. Chernomordik L. V. Zimmerberg J. (1997). Flickering fusion pores comparable with initial exocytotic pores occur in protein-free phospholipid bilayers. Proc. Natl. Acad. Sci. U.S.A. 94, 14423–14428. 10.1073/pnas.94.26.14423 9405628 PMC25008

[B6] Chernomordik L. V. Kozlov M. M. (2003). Protein-lipid interplay in fusion and fission of biological membranes. Annu. Rev. Biochem. 72, 175–207. 10.1146/annurev.biochem.72.121801.161504 14527322

[B7] Chernomordik L. V. Kozlov M. M. (2008). Mechanics of membrane fusion. Nat. Struct. Mol. Biol. 15, 675–683. 10.1038/nsmb.1455 18596814 PMC2548310

[B8] Dennison S. M. Bowen M. E. Brunger A. T. Lentz B. R. (2006). Neuronal SNAREs do not trigger fusion between synthetic membranes but do promote PEG-mediated membrane fusion. Biophys. J. 90, 1661–1675. 10.1529/biophysj.105.069617 16339880 PMC1367317

[B9] Devaux P. F. (1991). Static and dynamic lipid asymmetry in cell membranes. Biochemistry 30, 1163–1173. 10.1021/bi00219a001 1991095

[B10] Diao J. Cipriano D. J. Zhao M. Zhang Y. Shah S. Padolina M. S.. (2013). Complexin-1 enhances the on-rate of vesicle docking via simultaneous SNARE and membrane interactions. J. Am. Chem. Soc. 135, 15274–15277. 10.1021/ja407392n 24083833 PMC3854000

[B11] Diao J. Grob P. Cipriano D. J. Kyoung M. Zhang Y. Shah S.. (2012). Synaptic proteins promote calcium-triggered fast transition from point contact to full fusion. eLife 1:e00109. 10.7554/eLife.00109 23240085 PMC3514886

[B12] Diao J. Yoon T. Y. Su Z. Shin Y. K. Ha T. (2009). C2AB: a molecular glue for lipid vesicles with a negatively charged surface. Langmuir 25, 7177–7180. 10.1021/la901676e 19563216 PMC2730783

[B13] Essmann U. Perera L. Berkowitz M. L. Darden T. (1995). A smooth particle mesh ewald method. J. Chem. Phys. 103, 8577–8593. 10.1063/1.470117

[B14] Fesce R. Grohovaz F. Valtorta F. Meldolesi J. (1994). Neurotransmitter release: fusion or ‘kiss-and-run’? Trends Cell Biol. 4, 1–4. 10.1016/0962-8924(94)90025-6 14731821

[B15] Frolov V. A. Dunina-Barkovskaya A. Y. Samsonov A. V. Zimmerberg J. (2003). Membrane permeability changes at early stages of influenza hemagglutinin-mediated fusion. Biophys. J. 85, 1725–1733. 10.1016/S0006-3495(03)74602-5 12944287 PMC1303346

[B16] Gong B. Choi B.-K. Kim J.-Y. Shetty D. Ko Y. H. Selvapalam N.. (2015). High affinity host-guest FRET pair for single-vesicle content-mixing assay: observation of flickering fusion events. J. Am. Chem. Soc. 137, 8908–8911. 10.1021/jacs.5b05385 26160008

[B17] Graber Z. T. Gericke A. Kooijman E. E. (2014). Phosphatidylinositol-4,5-bisphosphate ionization in the presence of cholesterol, calcium or magnesium ions. Chem. Phys. Lipids 182, 62–72. 10.1016/j.chemphyslip.2013.11.004 24309195

[B18] Gramse G. Dols-Perez A. Edwards M. A. Fumagalli L. Gomila G. (2013). Nanoscale measurement of the dielectric constant of supported lipid bilayers in aqueous solutions with electrostatic force microscopy. Biophys. J. 104, 1257–1262. 10.1016/j.bpj.2013.02.011 23528085 PMC3602784

[B19] Gurtovenko A. A. Anwar J. Vattulainen I. (2010). Defect-mediated trafficking across cell membranes: insights from in silico modeling. Chem. Rev. 110, 6077–6103. 10.1021/cr1000783 20690701

[B20] Gurtovenko A. A. Vattulainen I. (2005). Pore formation coupled to ion transport through lipid membranes as induced by transmembrane ionic charge imbalance: atomistic molecular dynamics study. J. Am. Chem. Soc. 127, 17570–17571. 10.1021/ja053129n 16351063

[B21] He L. Wu L. G. (2007). The debate on the kiss-and-run fusion at synapses. Trends Neurosci. 30, 447–455. 10.1016/j.tins.2007.06.012 17765328

[B22] Helm C. A. Israelachvili J. N. McGuiggan P. M. (1992). Role of hydrophobic forces in bilayer adhesion and fusion. Biochemistry 31, 1794–1805. 10.1021/bi00121a030 1737032

[B23] Hess B. (2008). P-LINCS: a parallel linear constraint solver for molecular simulation. J. Chem. Theory. Comput. 4, 116–122. 10.1021/ct700200b 26619985

[B24] Humphrey W. Dalke A. Schulten K. (1996). VMD: Visual Molecular Dynamics. J. Mol. Graph. 14:33–38, 27–38. 10.1016/0263-7855(96)00018-5 8744570

[B25] Jackson M. B. Chapman E. R. (2008). The fusion pores of Ca2+ -triggered exocytosis. Nat. Struct. Mol. Biol. 15, 684–689. 10.1038/nsmb.1449 18596819 PMC2914174

[B26] Jahn R. Lang T. Sudhof T. C. (2003). Membrane fusion. Cell 112, 519–533. 10.1016/S0092-8674(03)00112-0 12600315

[B27] Jo S. Kim T. Iyer V. G. Im W. (2008). CHARMM-GUI: a web-based graphical user interface for CHARMM. J. Comput. Chem. 29, 1859–1865. 10.1002/jcc.20945 18351591

[B28] Jo S. Lim J. B. Klauda J. B. Im W. (2009). CHARMM-GUI Membrane Builder for mixed bilayers and its application to yeast membranes. Biophys. J. 97, 50–58. 10.1016/j.bpj.2009.04.013 19580743 PMC2711372

[B29] Jorgensen W. L. Chandrasekhar J. Madura J. D. Impey R. W. Klein M. L. (1983). Comparison of simple potential functions for simulating liquid water. J. Chem. Phys. 79, 926–935. 10.1063/1.445869

[B30] Klauda J. B. Venable R. M. Freites J. A. O'connor J. W. Tobias D. J. Mondragon-Ramirez C.. (2010). Update of the CHARMM all-atom additive force field for lipids: validation on six lipid types. J. Phys. Chem. B. 114, 7830–7843. 10.1021/jp101759q 20496934 PMC2922408

[B31] Kyoung M. Zhang Y. Diao J. Chu S. Brunger A. T. (2013). Studying calcium-triggered vesicle fusion in a single vesicle-vesicle content and lipid-mixing system. Nat. Protoc. 8, 1–16. 10.1038/nprot.2012.134 23222454 PMC3566647

[B32] Lai Y. Diao J. Cipriano D. J. Zhang Y. Pfuetzner R. A. Padolina M. S.. (2014). Complexin inhibits spontaneous release and synchronizes Ca^2+^-triggered synaptic vesicle fusion by distinct mechanisms. eLife 3:e03756. 10.7554/eLife.03756 25122624 PMC4130161

[B33] Lai Y. Diao J. Liu Y. Ishitsuka Y. Su Z. Schulten K.. (2013). Fusion pore formation and expansion induced by Ca^2+^ and synaptotagmin 1. Proc. Natl. Acad. Sci. U.S.A. 110, 1333–1338. 10.1073/pnas.1218818110 23300284 PMC3557091

[B34] Lai Y. Zhao L. Bu B. Lou X. Li D. Ji B.. (2015). Lipid molecules influence early stages of yeast SNARE-mediated membrane fusion. Phys. Biol. 12:025003. 10.1088/1478-3975/12/2/025003 25898400 PMC4827429

[B35] Leontiadou H. Mark A. E. Marrink S. J. (2004). Molecular dynamics simulations of hydrophilic pores in lipid bilayers. Biophys. J. 86, 2156–2164. 10.1016/S0006-3495(04)74275-7 15041656 PMC1304067

[B36] Li D. Ji B. Hwang K.-C. Huang Y. (2011). Strength of hydrogen bond network takes crucial roles in the dissociation process of inhibitors from the HIV-1 protease binding pocket. PLoS ONE 6:e19268 10.1371/journal.pone.0019268 21559397 PMC3084818

[B37] Li D. Ji B. Hwang K. Huang Y. (2010). Crucial roles of the subnanosecond local dynamics of the flap tips in the global conformational changes of HIV-1 protease. J. Phys. Chem. B. 114, 3060–3069. 10.1021/jp1005549 20143801

[B38] Li D. Liu M. S. Ji B. (2015). Mapping the dynamics landscape of conformational transitions in enzyme: the adenylate kinase case. Biophys. J. 109, 647–660. 10.1016/j.bpj.2015.06.059 26244746 PMC4572606

[B39] Marx V. (2014). A deep look at synaptic dynamics. Nature 515, 293–297. 10.1038/515293a 25391965

[B40] McLaughlin S. Murray D. (2005). Plasma membrane phosphoinositide organization by protein electrostatics. Nature 438, 605–611. 10.1038/nature04398 16319880

[B41] Müller M. Katsov K. Schick M. (2003). A new mechanism of model membrane fusion determined from Monte Carlo simulation. Biophys. J. 85, 1611–1623. 10.1016/S0006-3495(03)74592-5 12944277 PMC1303336

[B42] Parrinello M. Rahman A. (1981). Polymorphic transitions in single-crystals - a new molecular-dynamics method. J. Appl. Phys. 52, 7182–7190. 10.1063/1.328693

[B43] Richards D. A. (2009). Vesicular release mode shapes the postsynaptic response at hippocampal synapses. J. Physiol. 587, 5073–5080. 10.1113/jphysiol.2009.175315 19752123 PMC2790249

[B44] Risselada H. J. Grubmüller H. (2012). How SNARE molecules mediate membrane fusion: decent insights from molecular simulations. Curr. Opin. Struct. Biol. 22, 187–196. 10.1016/j.sbi.2012.01.007 22365575

[B45] Rizo J. Rosenmund C. (2008). Synaptic vesicle fusion. Nat. Struct. Mol. Biol. 15, 665–674. 10.1038/nsmb.1450 18618940 PMC2519048

[B46] Starai V. J. Jun Y. Wickner W. (2007). Excess vacuolar SNAREs drive lysis and Rab bypass fusion. Proc. Natl. Acad. Sci. U.S.A. 104, 13551–13558. 10.1073/pnas.0704741104 17699614 PMC1959418

[B47] Sun S. Wong J. T. Zhang T. Y. (2011). Atomistic simulations of electroporation in water preembedded membranes. J. Phys. Chem. B. 115, 13355–13359. 10.1021/jp206607j 21962234

[B48] Svennerholm L. (1968). Distribution and fatty acid composition of phosphoglycerides in normal human brain. *J*. Lipid Res. 9, 570–579. 4302302

[B49] Tieleman D. P. (2004). The molecular basis of electroporation. BMC Biochem. 5:10. 10.1186/1471-2091-5-10 15260890 PMC489962

[B50] Van Der Spoel D. Lindahl E. Hess B. Groenhof G. Mark A. E. Berendsen H. J. C. (2005). GROMACS: fast, flexible, and free. J. Comput. Chem. 26, 1701–1718. 10.1002/jcc.20291 16211538

[B51] Wang T. Smith E. A. Chapman E. R. Weisshaar J. C. (2009). Lipid mixing and content release in single-vesicle, SNARE-driven fusion assay with 1-5 ms resolution. Biophys. J. 96, 4122–4131. 10.1016/j.bpj.2009.02.050 19450483 PMC2712201

[B52] Weaver J. C. Chizmadzhev Y. A. (1996). Theory of electroporation: a review. Bioelectrochem. Bioenerget. 41, 135–160. 10.1016/S0302-4598(96)05062-3

[B53] Wong J. L. Koppel D. E. Cowan A. E. Wessel G. M. (2007). Membrane hemifusion is a stable intermediate of exocytosis. Dev. Cell 12, 653–659. 10.1016/j.devcel.2007.02.007 17420001 PMC1989768

[B54] Xu C. Li D. Cheng Y. Liu M. Zhang Y. Ji B. (2015). Pulling out a peptide chain from β-sheet crystallite: propagation of instability of H-bonds under shear force. Acta Mech. Sin. 31, 416–424. 10.1007/s10409-015-0404-y

[B55] Yang L. Huang H. W. (2002). Observation of a membrane fusion intermediate structure. Science 297, 1877–1879. 10.1126/science.1074354 12228719

[B56] Zhang Q. Li Y. Tsien R. W. (2009). The dynamic control of kiss-and-run and vesicular reuse probed with single nanoparticles. Science 323, 1448–1453. 10.1126/science.1167373 19213879 PMC2696197

[B57] Zhao W.-D. Hamid E. Shin W. Wen P. J. Krystofiak E. S. Villarreal S. A.. (2016). Hemi-fused structure mediates and controls fusion and fission in live cells. Nature 534, 548–552. 10.1038/nature18598 27309816 PMC4930626

[B58] Zucchi P. C. Zick M. (2011). Membrane fusion catalyzed by a Rab, SNAREs, and SNARE chaperones is accompanied by enhanced permeability to small molecules and by lysis. Mol. Biol. Cell 22, 4635–4646. 10.1091/mbc.E11-08-0680 21976702 PMC3226480

