# Supplementary material for: High Transmembrane Voltage Raised by Close Contact Initiates Fusion Pore
Source: Front Mol Neurosci. 2016 Dec 9;9:136. doi: 10.3389/fnmol.2016.00136 (PMC5145871; doi:10.3389/fnmol.2016.00136)
Supplement: Supplementary file 2 [file Presentation1.PDF]

Supplementary Material

High transmembrane voltage raised by close contact initiates fusion pore

Bing Bu[1,†], Zhiqi Tian[2,†], Dechang Li[1, *], and Baohua Ji[1, *]

[1]Biomechanics and Biomaterials Laboratory, Department of Applied Mechanics, Beijing Institute of Technology, Beijing 100081, China

[2]Center for Mitochondrial Biology and Medicine, The Key Laboratory of Biomedical Information Engineering of Ministry of Education, School of Life Science and Technology, Xi'an Jiaotong University, Xi'an 710049, China

[*]**Corresponding authors:** bhji@bit.edu.cn (BJ); dcli@bit.edu.cn (DL).

†These authors have contributed equally to this work.

Contents

1. Supplementary figures
2. Supplementary Equation 3
3. Supplementary movie - caption and snapshot

1. Supplementary figures

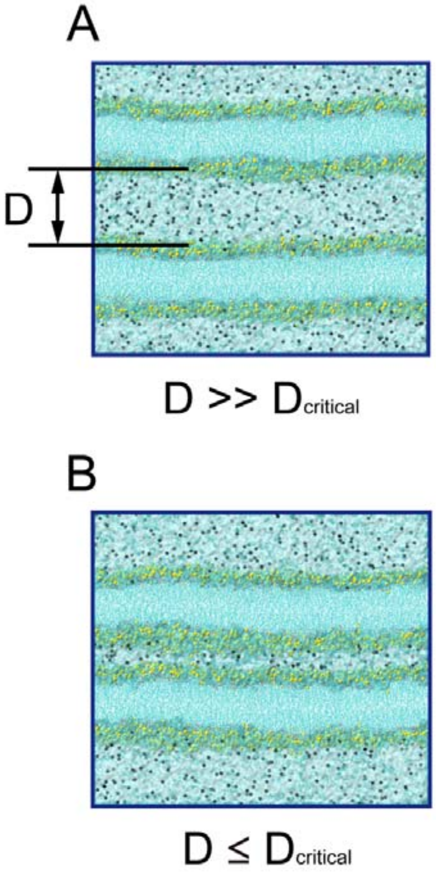

Figure S1. Simulation models of two apposed bilayers set at a series of given membrane distances D, where the distance is reduced from 6 nm with a step of 0.5 nm, for mimicking the approaching process of the two bilayers pulled by fusion proteins. Each simulation was carried out at a given constant membrane distance. The ion density is identical for this series of simulations at given lipid composition. Panel A and B illustrates two typical scenarios: **(A)** represents the situation when the two bilayers are relatively far away ($D >> D_{critical}$), where their interation is weak and there is no fusion pore formation; **(B)** represents the situation when the two bilayers are at close contact ($D \leqslant D_{critical}$), where there is pore formation.

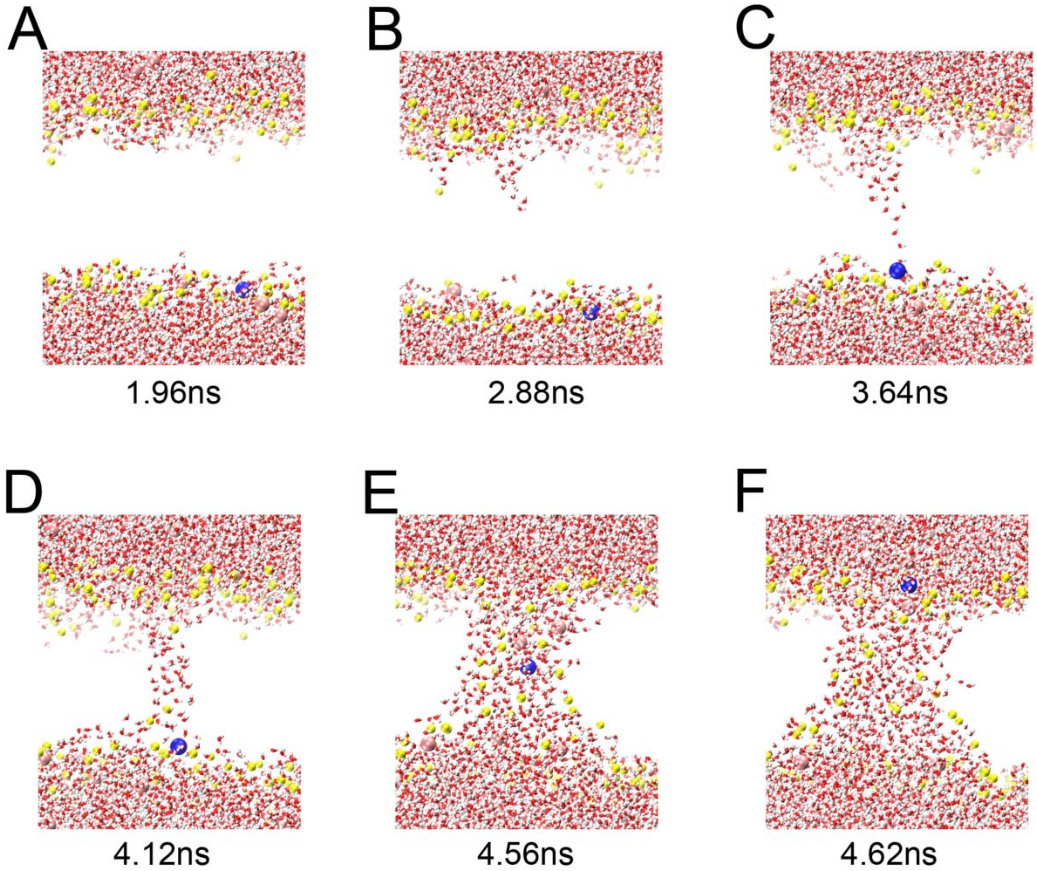

Figure S2. Process of transient pore formation. The tails of the lipids were not shown for a clear representation of the pore formation in the membrane. The phosphorus atoms in headgroups of lipids were shown by yellow dots. The water molecules were represented by red and white capsules. The blue dot represents the ion trafficking through the membrane.

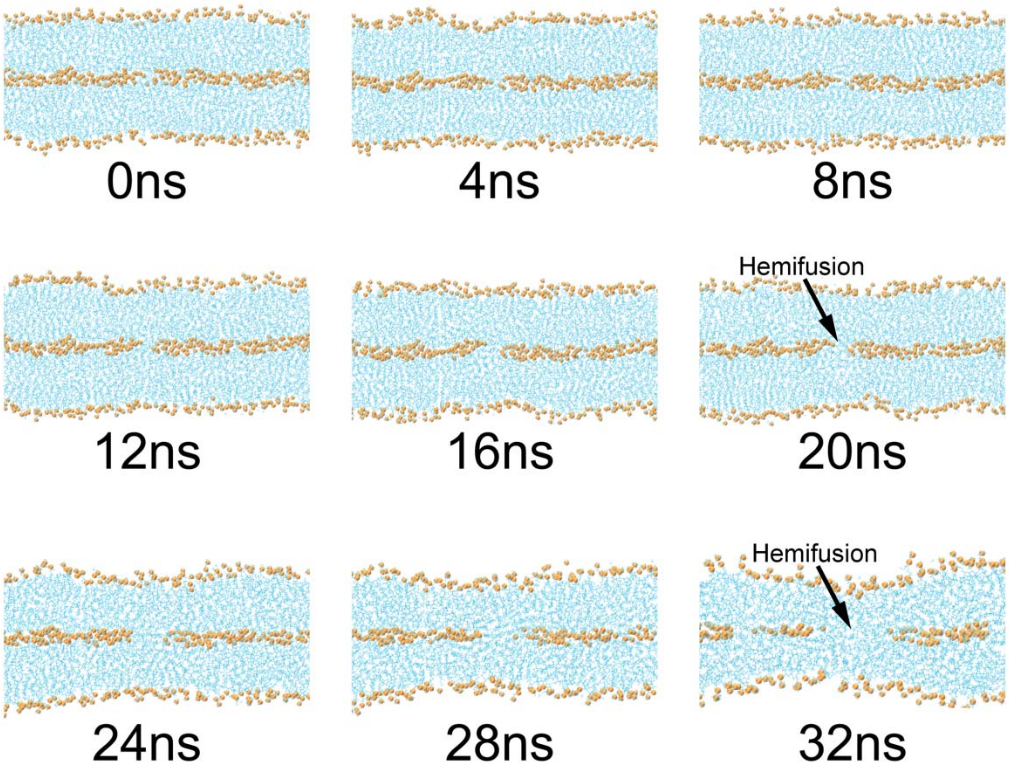

Figure S3. Formation of stalk-like structure for hemifusion when two membranes are in a much closer contact with a membrane distance smaller than the critical distance. The stalk-like structure initiated in a couple of nanoseconds (e.g. t=4ns) and formed in ~10-20ns. This result reproduced the experimental observation of formation of stalk-like structure under high dehydration condition (Yang and Huang, 2002). When a lateral force was applied (started from t=20ns), the stalk-like structure expanded and evolved towards a hemifusion structure.

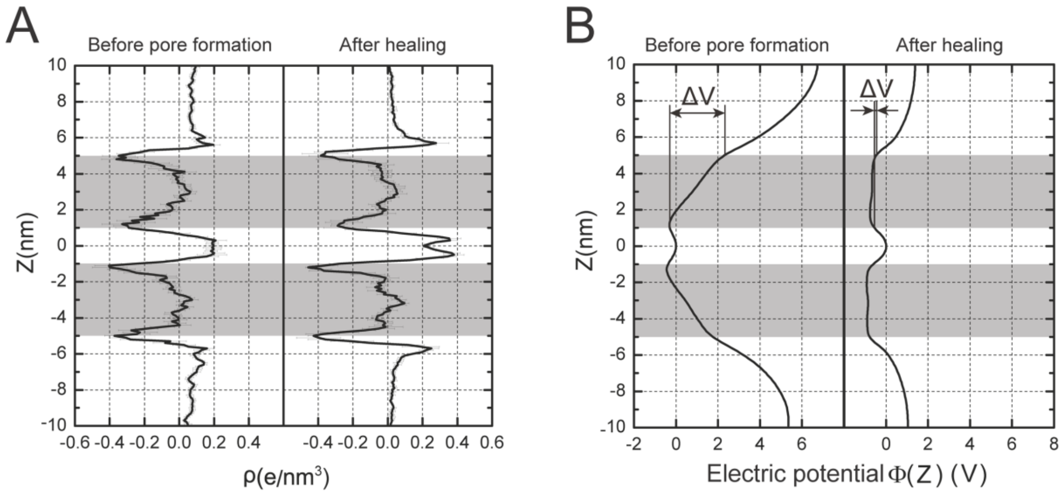

Figure S4. (A) The charge density distribution of the system calculated by MD simulations before pore formation and after pore closing, respectively. **(B)** Corresponding electric potential distribution before pore formation and after pore closing. The electric potential in MD simulations is calculated by adopting the procedure proposed by Tieleman by solving the one dimensional Poisson's equation (Tieleman, 2004).

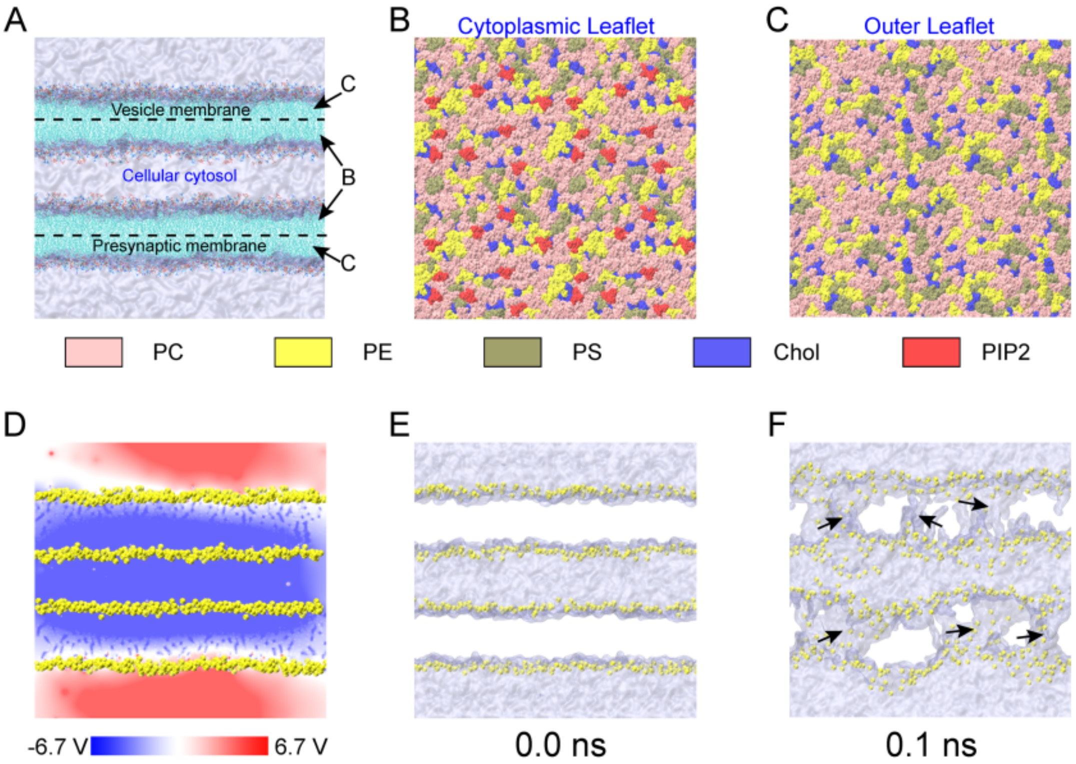

Figure S5. Transient water pore formation in the asymmetric membranes. (A) The illustration of membranes with asymmetric lipid composition in the side view. (**B**) The lipid composition of the cytoplasmic leaflet, Chol : DOPC : POPE : POPS : PIP2 = 20% : 45% : 20% : 10% : 5%. (**C**) The lipid composition of the outer leaflet, Chol : DOPC : POPE : POPS = 20% : 45% : 20% : 15%. For clarify, different types of lipids are represented by different color in (**B**) and (**C**). (**D**) The electric potential field at t=0ns. High transmembrane voltage was induced by the close contact of the two membranes. (**E**) The initial structure at t=0 ns and (**F**) The transient pores occurred at t=0.1ns.

2. Supplementary Equation S1

The results of the integral of Eq. (3) can be derived as

$$\Phi(Z) = -\frac{1}{\varepsilon}\int_{-(H_m+\frac{D}{2})}^{Z}\int_{-(H_m+\frac{D}{2})}^{Z'}\rho(Z'')\,dZ''dZ'$$

$$= -\frac{1}{\varepsilon}\times\begin{cases}
\frac{1}{2}\rho_3 H_L^2 - \frac{1}{2}\rho_{m2}H_m^2 + \frac{1}{2}\rho_3 DH_L + \frac{1}{2}\rho_{m2}DH_m + \rho_3 H_m H_L + \rho_{m2}H_m^2 - \\
\quad \frac{1}{2}\rho_{m1}H_m^2 + \frac{\rho_1 D^2}{8} + \frac{1}{2}\rho_1 DH_m + \frac{1}{2}\rho_1 H_m^2 - \frac{1}{2}\rho_{m1}DH_m + & Z \in R_1 \\
\quad \frac{1}{2}\rho_1 Z^2 + (\rho_3 H_L + \rho_{m2}H_m + \rho_2 D + \rho_{m1}H_m - \rho_1 H_m - \frac{\rho_1 D}{2})Z \\
\\
\frac{1}{2}\rho_3 H_L^2 + \frac{1}{2}\rho_{m2}H_m^2 + \frac{1}{2}\rho_3 DH_L + \frac{1}{2}\rho_{m2}DH_m + \rho_3 H_m H_L - \frac{1}{2}\rho_2 D^2 - \frac{1}{8}\rho_{m1}D^2 + \frac{\rho_{m1}D^2}{4} + & Z \in R_{m2} \\
\quad \frac{1}{2}\rho_{m1}Z^2 + (\rho_3 H_L + \rho_{m2}H_m + \rho_2 D - \frac{\rho_{m1}D}{2})Z \\
\\
\frac{1}{2}\rho_3 H_L^2 + \frac{1}{2}\rho_{m2}H_m^2 + \frac{1}{2}\rho_3 DH_L + \frac{1}{2}\rho_{m2}DH_m + \rho_3 H_m H_L + & Z \in R_2 \\
\quad \frac{1}{8}\rho_2 D^2 + \frac{1}{2}\rho_2 Z^2 + (\rho_3 H_L + \rho_{m2}H_m + \frac{\rho_2 D}{2})Z \\
\\
\frac{1}{2}\rho_3 H_L^2 + \frac{1}{2}\rho_{m2}H_m^2 + \frac{1}{2}\rho_3 DH_L + \frac{1}{2}\rho_{m2}DH_m + \rho_3 H_m H_L + \frac{1}{8}\rho_{m2}D^2 + & Z \in R_{m1} \\
\quad \frac{1}{2}\rho_{m2}Z^2 + (\rho_3 H_L + \rho_{m2}H_m + \frac{\rho_{m2}D}{2})Z \\
\\
\frac{1}{2}\rho_3 H_L^2 + \frac{1}{2}\rho_3 DH_L + \rho_3 H_m H_L + \frac{1}{2}\rho_3 H_m^2 + \frac{1}{8}\rho_3 D^2 + \frac{1}{2}\rho_3 DH_m + & Z \in R_3 \\
\quad \frac{1}{2}\rho_3 Z^2 + (\rho_3 H_L + \rho_3 H_m + \frac{\rho_3 D}{2})Z
\end{cases}$$

where ρ_1, ρ_2, ρ_3, ρ_{m1} and ρ_{m2} are the charge density in layers R_1, R_2, R_3, R_{m1}, and R_{m2}, respectively. H_L is the thickness of domain R_1 and R_3, H_m is the thickness of the lipid bilayer of membranes, and *D* is the inter-membrane distance.

3. Supplementary movies

Movie S1. Movie of the sequential processes of nano-pore formation, ions transportation and pore healing. The movie is submitted as a separate file. The full trajectory XTC files can be also provided up requested.

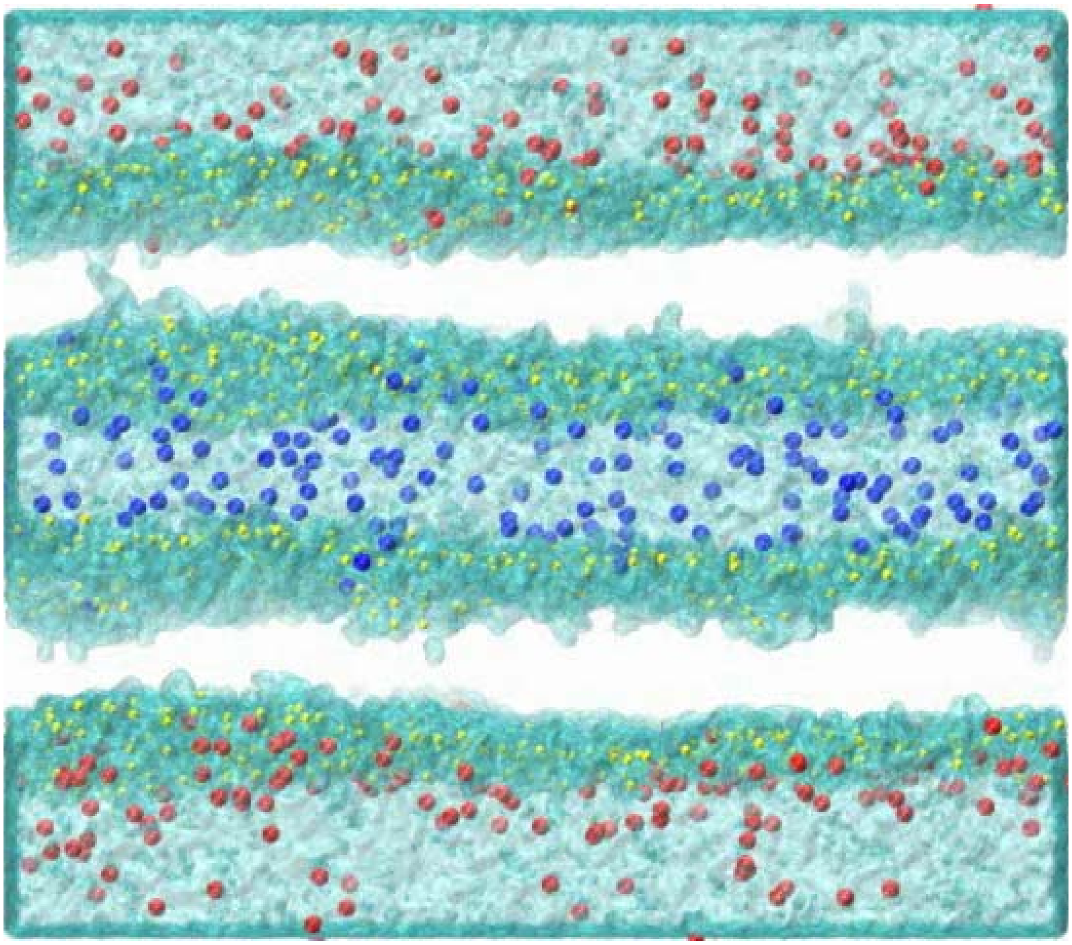

References

Tieleman, D.P. (2004). The molecular basis of electroporation. *BMC Biochem* 5**,** 10. doi: 10.1186/1471-2091-5-10.

Yang, L., and Huang, H.W. (2002). Observation of a membrane fusion intermediate structure. *Science* 297**,** 1877-1879. doi: 10.1126/science.1074354.
